# Tunable Oxygen Connectivity in Carbaporphyrinoid Polymers on Metal Surfaces

**DOI:** 10.1002/anie.9635327

**Published:** 2026-08-05

**Authors:** Alberto Martínez‐Bajo, Maxence Urbani, Ana Barragán, Óscar Jover, Elena Pérez‐Elvira, Ilaria Tomei, Claudio Goletti, Alejandro Jiménez‐Martín, Aurelio Gallardo, Koen Lauwaet, David Écija, Diego Soler‐Polo, Marco Di Giovannantonio, Tomás Torres, José I. Urgel

**Affiliations:** ^1^ IMDEA Nanoscience, C/ Faraday 9 Campus De Cantoblanco Madrid Spain; ^2^ CNR – Istituto di Struttura della Materia (CNR‐ISM) Roma Italy; ^3^ Dipartimento Di Fisica University of Rome “Tor Vergata” Roma Italy; ^4^ Unidad De Nanomateriales avanzados, Imdea Nanoscience Unidad Asociada Al CSIC Por El ICMM Madrid Spain; ^5^ Departamento De Química Orgánica and Institute for Advanced Research in Chemistry (IAdChem) Universidad Autónoma de Madrid Madrid Spain

**Keywords:** carbaporphyrinoids, metal–organic networks, nc‐AFM, oxygen‐based reactions, porphyrinoid polymers, scanning tunneling microscopy, XPS

## Abstract

Controlling oxygen‐mediated connectivity at metal surfaces offers a powerful route to engineer covalent and metal–organic architectures with atomic precision. Here, we demonstrate tunable oxygen‐based connectivity in carbaporphyrinoid polymers on Au(111) under ultrahigh vacuum (UHV) conditions. Initial thermal activation of hydroxyl‐functionalized dicarbahemiporphyrazine precursors induces carbon–carbon coupling via a [3+3] cycloaromatization, affording one‐dimensional polymers equipped with hydroxyl‐terminated units. From this intermediate two distinct oxygen‐based linkages can be achieved: further annealing induces dehydrogenative coupling of adjacent polymers through the hydroxyls groups to yield dibenzo‐p‐dioxane (O‐heterocyclic) bridges that covalently connect the carbaporphyrinoid macrocycles, whereas dosing cobalt produces extended metal–organic networks stabilized by two‐fold O···Co···O coordination motifs accompanied by cobalt‐metalated macrocycles. Scanning tunneling microscopy (STM) and non‐contact atomic force microscopy (nc‐AFM) directly resolve both the coordinated networks and the O‐heterocyclic bridging motifs. Complementary DFT and Free energy calculations elucidate the reaction pathways, while x‐ray photoelectron spectroscopy (XPS) reveals characteristic chemical shifts that account for C–O–C formation and suggest Co···O coordination. This controllable oxygen‐based connectivity provides a versatile platform to tune structure and electronic properties in porphyrinoid‐based polymers.

## Graphical abstract

Hydroxyl‐functionalized dicarbaporphyrinoid polymers synthesized on Au(111) enable tunable oxygen‐mediated connectivity under UHV conditions. Thermal activation yields one‐dimensional hydroxyl‐terminated polymers that can selectively evolve into either covalently‐linked dibenzo‐p‐dioxane architectures through intermolecular C–O–C coupling or two‐fold Co‐coordinated metal–organic networks. STM, nc‐AFM, XPS, and DFT reveal the structural motifs, reaction pathways, and chemical fingerprints governing these divergent on‐surface transformations.

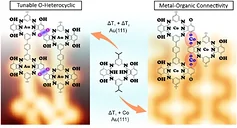

## 1 Introduction

Porphyrinoids constitute a broad class of heteroaromatic macrocycles that have been central to both fundamental chemistry and applied sciences [1]. Their unique electronic structures and extended π‐conjugation provide them with remarkable optical, redox, and coordination properties, which underpin their essential roles in nature. Beyond biology, synthetic porphyrinoids have long been exploited in analytical chemistry [2], molecular recognition [3], photocatalysis [4], light‐harvesting [5], nonlinear optics [6], and organic electronics [7], owing to their tunability and stability. Despite the extensive investigation of porphyrinoid derivatives, carbaporphyrinoids, in which one or more pyrrolic nitrogens are substituted by carbon atoms, have received relatively little attention, making them a compelling target for exploring new reactivity and functionality [8, 9, 10]. This modification disrupts the conventional 18 π‐electron aromaticity, giving rise to new resonance structures and non‐traditional aromatic character with altered electronic configurations. As a result, carbaporphyrinoids display unusual reactivity and broad redox activity while maintaining the versatility of the porphyrinoid scaffold for further functionalization [11, 12, 13]. Their ability to host substituents at both the macrocyclic core and the periphery makes them promising building blocks for conjugated molecular architectures, where their electronic properties can be coupled with tailored supramolecular interactions.

Studying porphyrinoids adsorbed on surfaces is particularly appealing as it allows precise control over their adsorption geometry, electronic coupling to the substrate, and intermolecular interactions; factors that are critical for tuning their functionality [14, 15]. In addition, the use of scanning probe microscopies provides direct atomic‐scale insight into their structure, reactivity, and electronic states, enabling correlations between molecular design and emergent properties that are difficult to study in solution‐phase chemistry.

Metallo‐supramolecular engineering [16, 17, 18] and, more recently, the on‐surface synthesis of covalent porphyrinoid nanoarchitectures [14] directly on surfaces have enabled the precise design of functional molecular materials with tailored electronic, magnetic, and catalytic properties [19, 20, 21, 22, 23, 24, 25, 26, 27, 28, 29]. However, on‐surface studies of many interfacial metallo‐supramolecular designs [30], together with fundamental chemical reactions [31, 32] with important implications in organic synthesis have still remained scarce. In this context, hydroxyl functionalities represent particularly versatile functional groups for surface chemistry, as they can undergo selective dehydrogenation, condensation, or ring‐forming reactions, while also serving as coordination sites for metal centers. Such dual reactivity opens interesting pathways to engineer porphyrinoid‐based polymers and networks in which covalent O‐heterocyclic linkages coexist or compete with metal–oxygen coordination motifs, thereby enabling control over connectivity, dimensionality, and functionality at surfaces [33, 34, 35, 36, 37].

In this study, we highlight the conceptual ability of hydroxyl‐functionalized dicarbahemiporphyrazine (dcHp) precursors to direct a single polymeric intermediate toward distinct structural outcomes, namely surface‐confined covalent coupling and coordination architectures, on a metal substrate under ultra‐high vacuum (UHV) conditions. As anticipated in Scheme 1, we demonstrate that controlled thermal activation of such precursors triggers a [3+3] cycloaromatization, yielding one‐dimensional polymers with hydroxyl‐terminated edges. This intermediate serves as a versatile platform from which two oxygen‐based connectivity motifs can be selectively accessed. Further annealing induces the dehydrogenative coupling between neighboring hydroxyl groups of adjacent polymers to form dibenzo‐p‐dioxane bridges, generating covalently linked porphyrinoid polymers. Alternatively, deposition of cobalt onto the intermediate induces the formation of extended metal–organic networks, stabilized by two‐fold O**···**Co**···**O coordination and metalation of the dicarbahemiporphyrazine macrocycle. Using high‐resolution scanning tunneling microscopy (STM) and non‐contact atomic force microscopy (nc‐AFM), we resolve both the O‐heterocyclic and Co‐coordinated architectures at the molecular level. Complementary, density functional theory (DFT) calculations provide insight into the mechanisms underlying these transformations, while x‐ray photoelectron spectroscopy (XPS) confirms the chemical nature of the resulting linkages. This dual strategy highlights the potential of oxygen‐mediated surface chemistry to tune the structural and electronic properties of carbaporphyrinoid‐based polymers, providing a basis for extending such reactivity to other molecular scaffolds.

**SCHEME 1:**
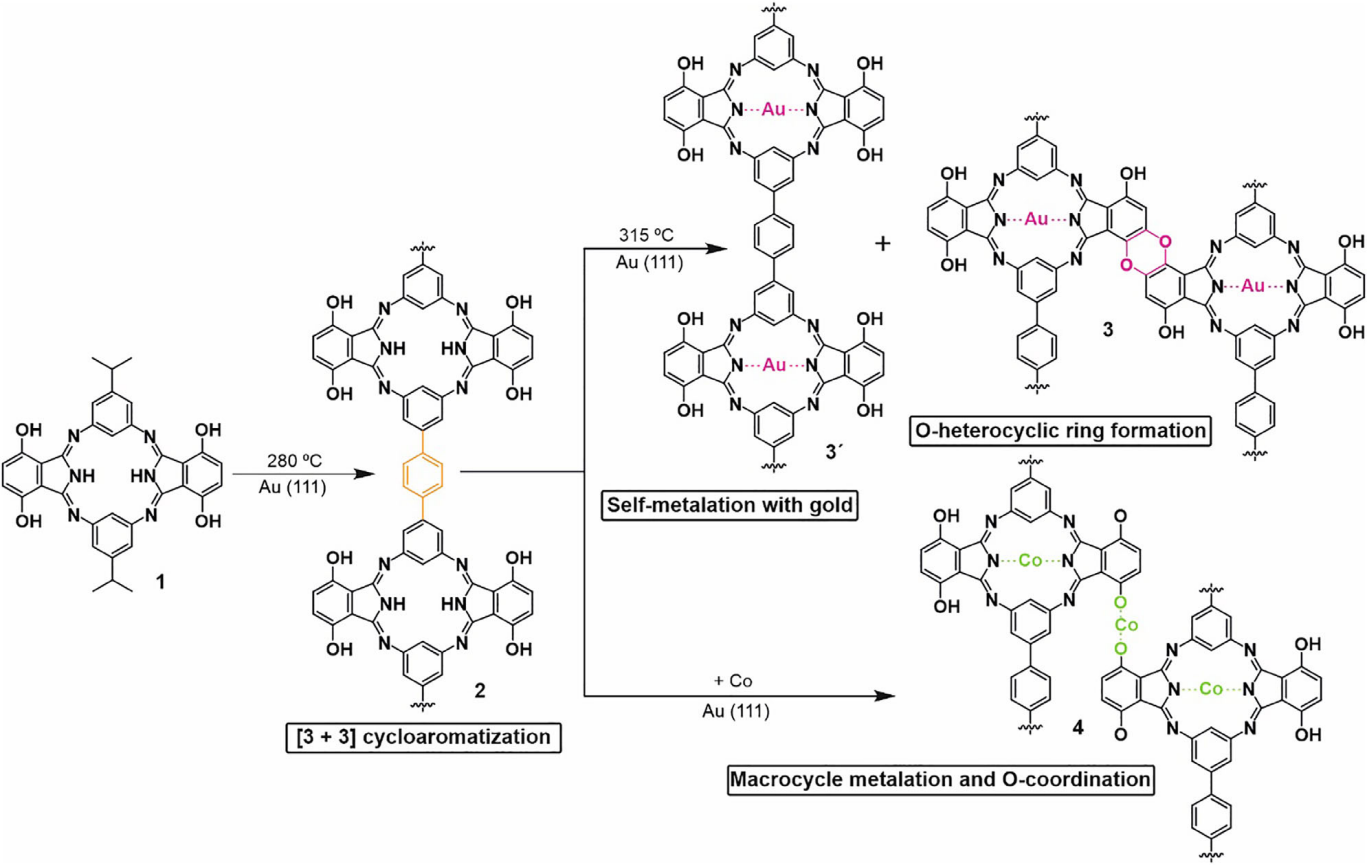
On‐surface reaction schemes towards the synthesis, coordination and metalation of low‐dimensional dcHp‐based polymers.

## 2 Results and Discussion

### 2.1 On‐surface Polymerization, Gold Self‐Metalation and Covalent Interchain Coupling of Hydroxyl‐Functionalized Dicarbahemiporphyrazines

Prior to any on‐surface deposition, **1** was synthesized by conventional solution chemistry, which has purposely been designed with two opposing isopropyl groups at the benzene positions, as well as two hydroxyl groups at the m,m’‐positions of the two opposite isoindole units. A condensation reaction of 5‐isopropylbenzene‐1,3‐diamine and 2,3‐dicyanobenzoquinone in refluxing ethane‐1,2‐diol for 24 h afforded dcHp **1** as a dark brown‐black solid in a remarkable high yield (quantitative). It must be noticed that **1** is insoluble in all common solvents (i.e.: alcohol, CHCl_3_, DMSO, THF, or toluene). Fortunately, the tetra sodium salt of dcHp‐**1** [**1** ^4−^, 4Na^+^] formed in‐situ upon addition of NaOH in MeOH or DMSO solutions (deeply blue‐colored) presents a high solubility, which enabled its full characterization by ^1^H‐ and ^13^C‐ NMR and UV–visible spectroscopies in solution (showing well‐defined spectra), as well as by high‐resolution mass spectrometry (see Figure S1–S8)).

To investigate the on‐surface reactions depicted in Scheme 1, a submonolayer coverage of **1** was sublimed intact onto an Au(111) substrate held at room temperature and subsequently annealed to 280°C to induce surface‐catalyzed [3+3] cycloaromatization of the monomers, yielding 1D polymers connected through benzene rings. This chemical reaction has recently been employed on gold surfaces to covalently link several carbon‐based nanostructures [24, 38, 39, 40, 41, 42]. Figure S9 shows an overview constant‐current and constant‐height STM images of the sample after the annealing step, revealing the formation of the polymer chains (**2**) distributed across the Au(111) surface. Most of the macrocycles are freebase with chains being aligned into ordered assemblies stabilized by intermolecular O···H interactions between adjacent units with a projected distance of 2.3 ± 0.5 Å [43]. While long‐range ordered polymers have been achieved using less complex precursors and coupling motifs, the present system relies on a multifunctional hemiporphyrazine scaffold incorporating isopropyl and hydroxyl groups, which introduces increased steric constraints and limits surface diffusion. As a result, the achievable polymer length and long‐range order are inherently restricted [38]. In addition, the polymer terminations do not follow a well‐defined motif, often displaying the detachment of isopropyl substituents or structural irregularities associated with non‐regular couplings between isopropyl and hydroxyl functional groups. Further annealing to 315°C (Figure 1a) gives rise to isolated chains where the macrocycles have been self‐metalated with Au atoms (**3`**) coexisting with patches of covalently coupled nanostructures (**3**), Figure 1b displays an STM image of a polymer chain composed of three dcHp units. The corresponding nc‐AFM image acquired with a CO‐functionalized tip unambiguously confirms the chemical structure of **3’**. Figure 1c depicts the resulting constant‐height frequency‐shift image where individual benzene rings linking dcHps, the four hydroxyl functional groups per molecule and the self‐metalation of the macrocycle by gold adatoms can be clearly resolved, providing direct evidence of the of the successful formation of polymer **3’** and the preservation of its functional moieties. Such experimental features, observed in Figure 1c, are well reproduced by the DFT‐optimized geometry and the corresponding nc‐AFM simulation of **3’** on the Au(111) surface (Figure 1d). Notably, within the same sample we identify in the acquired STM images small patches where adjacent chains are covalently interconnected (Figure 1e). These nanostructures can be attributed to the formation of unprecedented intermolecular O‐heterocyclic motifs (**3**), corresponding to dioxin rings, generated through dehydrogenative C–O–C couplings between hydroxyl groups of neighboring polymers (a statistical analysis of the chain‐length distributions for each species is provided in Figure S10). Whereas intramolecular C–O–C closures have been recently reported [36, 44, 45, 46], this constitutes to the best of our knowledge, the first on‐surface realization of an intermolecular covalent coupling in which hydroxyl groups act as direct precursors of the bridging oxygen atoms in the final structure.

**FIGURE 1:**
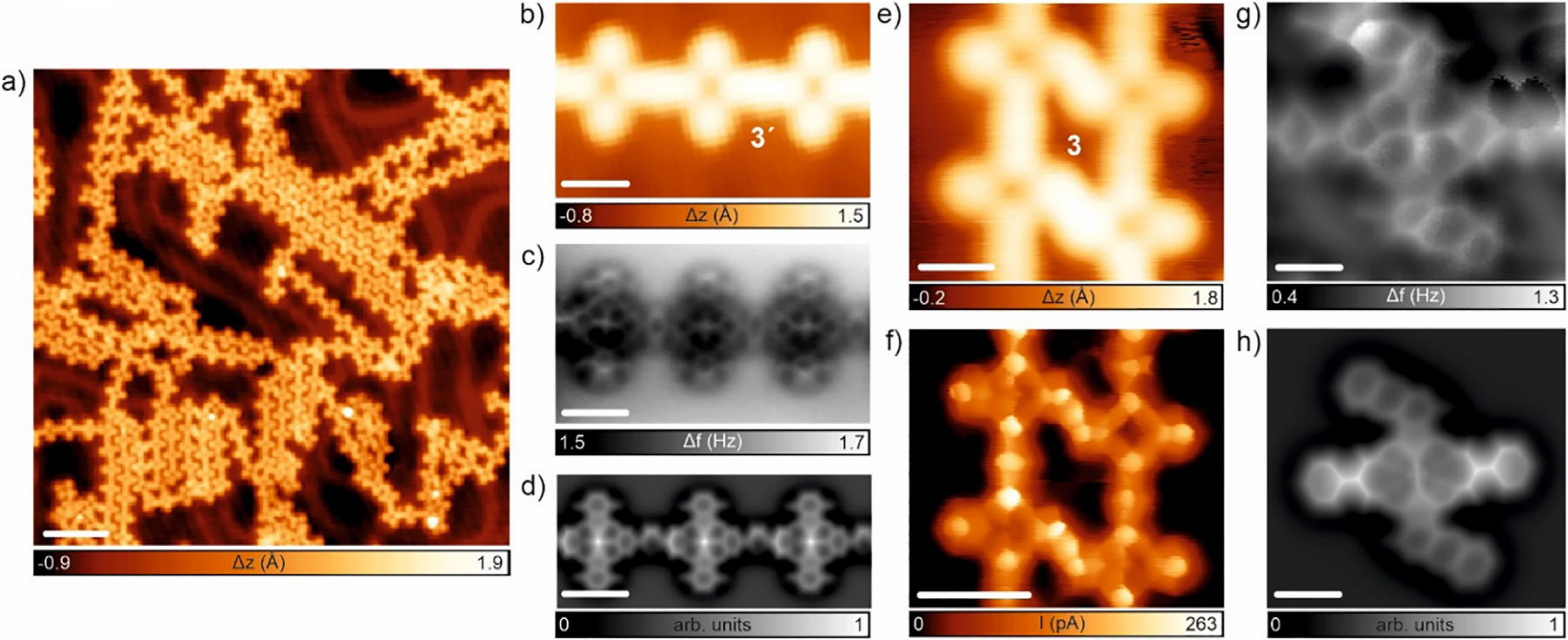
O‐heterocyclic bridging of hydroxyl‐functionalized dcHp polymers on Au(111). (a) Overview STM image of the Au(111) surface after deposition of **1** and subsequent annealing at 315°C. V_b_ = 400 mV, I_t_ = 50 pA, scale bar: 5 nm. (b) High‐resolution STM image of an isolated segment of polymer **3’**. V_b_ = 500 mV, I_t_ = 50 pA, scale bar: 0.9 nm. (c and d) Constant‐height frequency shift nc‐AFM and simulated nc‐AFM images of three gold self‐metalated dcHp units of polymer **3’**. Z‐offset 160 pm below STM set point: 5 mV, 50 pA, scale bar: 0.9 nm. (e and f) Constant‐current and constant‐height zoom‐in STM image of a small patch of **3** composed of four dcHp units. (e) V_b_ = 500 mV, It = 50 pA, scale bar: 0.9 nm. (f) V_b_ = 0.5 V, I_t_ = 50 pA, scale bar: 1.2 nm. (g and h) Constant‐height frequency shift and simulated nc‐AFM images of the gold self‐metalated dcHp unit shown in (f), respectively. Z‐offset 190 pm below STM set point: 5 mV, 50 pA. Scale bar: 0.5 nm. All the experimental SPM images shown in this figure have been acquired with a CO‐functionalized tip.

### 2.2 Reaction Pathway Toward Intermolecular O‐Heterocyclic Coupling

To understand the reaction mechanism that leads to the formation of the O‐heterocyclic bond, we have performed a series of DFT simulations for a model system on surface at PBE level of theory [47]. We study the relative stability of the initial, final and intermediate states indicated in Figure 2, as well as the energies of the transition states found by means of the NEB method [48]. In the proposed reaction mechanism, the hydroxyl groups are initially dehydrogenated by nearby gold adatoms, leading to the formation of the intermediate states **I1** and **I2**. From **I2**, we explore the formation of each C─O─C bond, in which a nearby gold adatom assists the dehydrogenation of the closest carbon atom of the adjacent molecule. Thus we reach the intermediate state **I3** (one C─O─C bond formed) and the final state (**FS**, where the full cycle is achieved). Interestingly, in order to predict the correct thermodynamic character of the reaction, it is necessary to include the entropic effects of the evaporated hydrogen atoms at high temperatures. For this purpose, vibrational energy and entropy contributions from both the molecules and the abstracted hydrogen atoms were added to the DFT‐calculated energies The leading term in this correction is the entropy of the hydrogens, which contributes by ‐1.01 eV each [49]. The adatoms play an important role in lowering the energy barriers and thus facilitating the reaction kinetics. The bottleneck of the reaction, **I1** – **TS2** (see Figure 2), happens in a matter of minutes for temperatures in a range from 300 to 350°C, according to Eyring's equation, which renders the reaction feasible [50].

**FIGURE 2:**
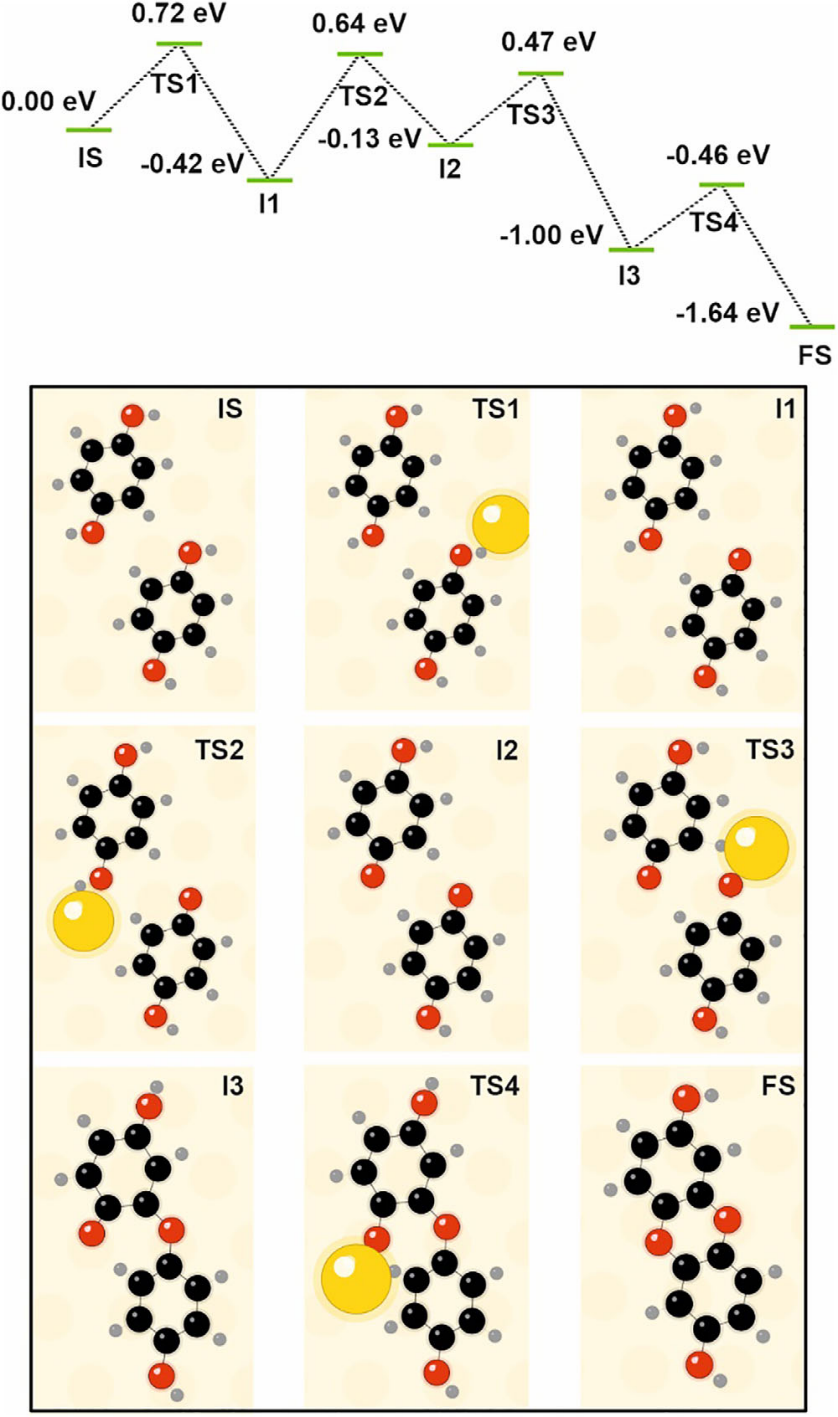
Free energy profile at *T* = 300 K for the proposed reaction mechanism. Adatom‐assisted dehydrogenation yields I1 and I2, where overall a H_2_ molecule is evaporated and the C─O bonds become double bonds (C = 0). Then, on each side, the C─O bond is formed and another hydrogen is lost. Carbon atoms are depicted in black, nitrogen in light blue, oxygen in red, hydrogen in grey, and gold in yellow.

### 2.3 Metal–Organic Coordination and Metalation of Carbaporphyrinoid Polymers

After addressing the hydroxyl‐driven intermolecular O‐heterocyclic coupling, we studied the interaction of polymer **2** with Co atoms. Figure 3a shows an overview constant‐current STM image of a sample containing **2** and co‐deposited cobalt atoms (see Methods for details on the sample preparation). Clearly, the polymers assemble now into ordered arrays, where adjacent polymers are held at a uniform separation (Figure 3b). We attribute this regular spacing to a two‐fold O···Co···O coordination, in which cobalt atoms bridge neighboring polymers with a projected Co···O bond length equal to 1.9 ± 0.1 Å [33, 43], thereby enforcing a well‐defined interchain distance and giving rise to relatively extended metal–organic networks on the surface (see Figure S11 where nc‐AFM images of polymer chains before and after Co deposition are shown). In addition, constant‐current (Figure 3b) and constant‐height (Figure 3c) STM images reveal bright protrusions centered at the dcHp macrocycles. These zero‐bias features are absent in free‐base dcHp macrocycles and attributed to a Kondo resonance state due to the Co‐induced metalation of the macrocyclic cores (Figure S12) [38, 51], resulting from the coexistence of O···Co···O coordination architectures and metalated dicarbaporphyrinoids (**4**) on the Au(111) surface. Importantly, lateral coordination and metalation occur simultaneously at the scanning conditions (∼4 K), with neither process dominating over the other. Single transition metal and lanthanide atoms and oxygen containing organic ligands are usually coordinated in a 3‐fold and higher geometry on a metal surface [33, 34, 35, 52, 53, 54]. However, in our case, the coordination is limited to a two‐fold O···Co···O geometry, most likely imposed by steric constraints and the rigid arrangement of the polymer backbones, which prevent the metal center from achieving higher coordination number.

**FIGURE 3:**
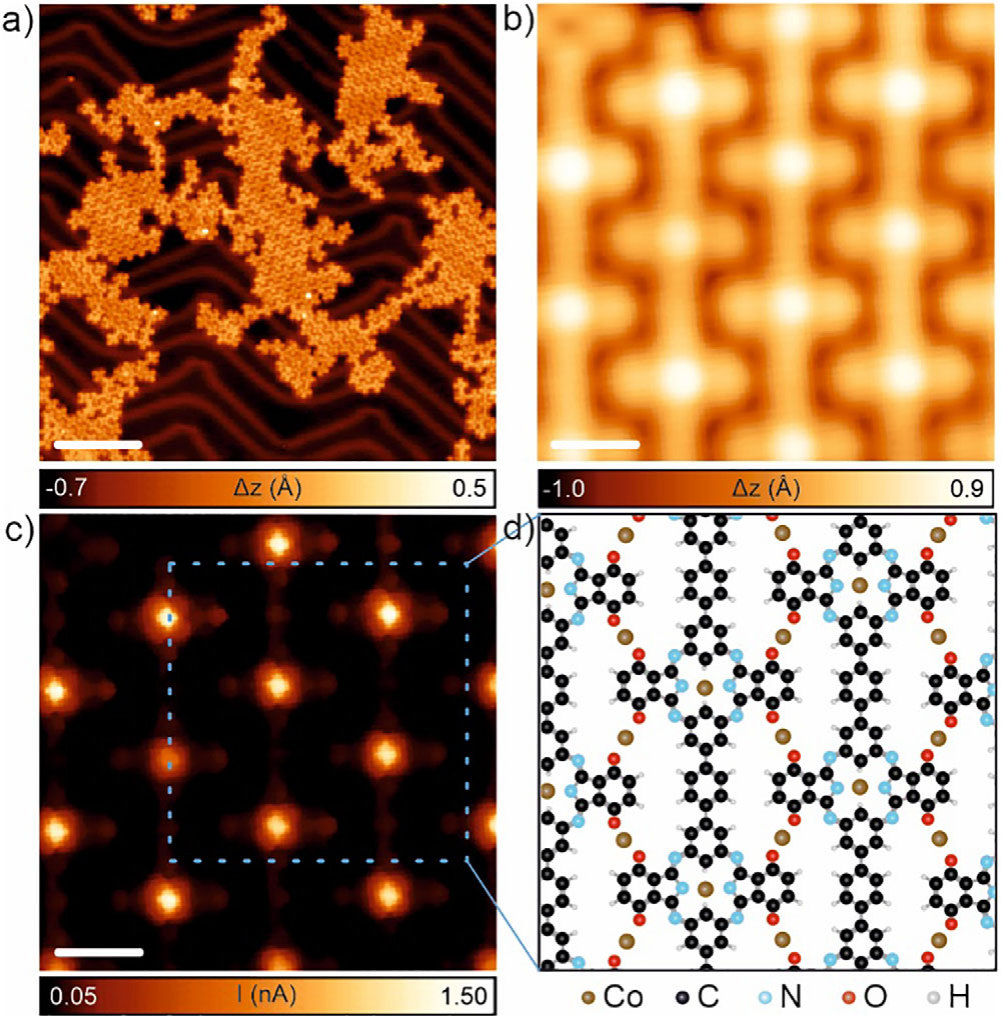
Co coordination and metalation of polymer **2** on Au(111). (a) Overview STM image of the Au(111) surface after deposition of a flux of Co atoms on a sample containing polymer **2**. Several network domains are observed. V_b_ = ‐500 mV, I_t_ = 100 pA, scale bar: 10 nm. (b) and (c) High‐resolution constant‐current and constant‐height STM images of **4**. (b) V_b_ = 50 mV, I_t_ = 50 pA, scale bar: 1.0 nm. (c) Z‐offset 175 pm below STM set point V_b_ = 5 mV, I_t_ = 50 pA, scale bar: 1.0 nm. (d) Corresponding structural model highlighting the coordination network and metalation by Co atoms shown in (b and c).

### 2.4 XPS Investigation of Thermal Reactivity and Cobalt‐Assisted Transformations

Finally, to confirm the binding motifs observed upon stepwise annealing, we performed XPS measurements of the systems under investigation at different temperatures. Figure 4 reports an overview of the results, which include temperature‐programmed (TP) and high‐resolution (HR) XPS data for the system with and without co‐deposited cobalt atoms. All samples had a molecular coverage between 0.25 and 0.35 monolayers (ML) of molecule **1** on Au(111). We first monitored the temperature evolution during annealing of the molecules from RT to 550°C, achieved with a constant heating rate of 4°C min^−1^ (TP‐XPS maps in Figures 4a,b). Two main spectral features are observed for both N 1s and O 1s after RT deposition of **1** on Au(111). Figure 4m,n shows the deconvolution of the corresponding HR‐XPS spectra. O 1s is expected to have only one component, associated to the ─OH chemical state. However, two components are observed at 530.8 and 532.9 eV. While the latter appears at the binding energy (BE) expected for ─OH, the former is at a BE value typical of C═O groups [55]. Hence, we interpret this as a partial dehydrogenation of the hydroxyl groups upon adsorption of **1** on Au(111). N 1s presents two main components at 397.8 and 400.0 eV, associated to the iminic (─N═) and aminic (NH) nitrogen atoms, respectively [56]. Moreover, two shoulders are visible at higher BE values, which we assign to the interaction of the molecules with gold adatoms from the substrate, as reported elsewhere [57, 58]. The fact that the aminic component is more intense than the iminic one, counterintuitive based on the molecular design, is attributed to the interaction of some of the periphery nitrogen atoms with the H atoms that are being detached from the hydroxyl groups, which renders them more of aminic character (see Figure S13 for the ab initio free molecular dynamics showing the quasi‐metastable states in intermediate positions for the H). This chemical state remains nearly unaltered until 350°C, where we start observing an evolution of the signals (Figure 4a,b). HR‐XPS spectra acquired after annealing the sample at 490°C (see Methods for details on the annealing procedures and reported temperature values) reveal in N 1s the expected self‐metalation of the molecular core with Au atoms from the surface (peak at 398.4 eV in Figure 4e) [56], and in O 1s the appearance of two new components at 531.5 eV and 533.8 eV, accompanied by a significant reduction of the other two components. We assign the new O 1s component at 531.5 eV to the dehydrogenated O atoms interacting with Au adatoms from the surface, and the one at 533.8 eV to the O‐heterocyclic ring formation with C–O–C binding motifs [46]. The extra component observed in the N 1s signal at 400.2 eV can be attributed to a graphitic nitrogen chemical environment [59] due to a partial lateral fusion of the polymeric chains occurring at such high annealing temperature. Therefore, these results confirm the self‐metalation with gold atoms from the surface (starting at 350°C and terminating at 500°C) and the achievement of the O‐heterocyclic rings (at about 500°C).

**FIGURE 4:**
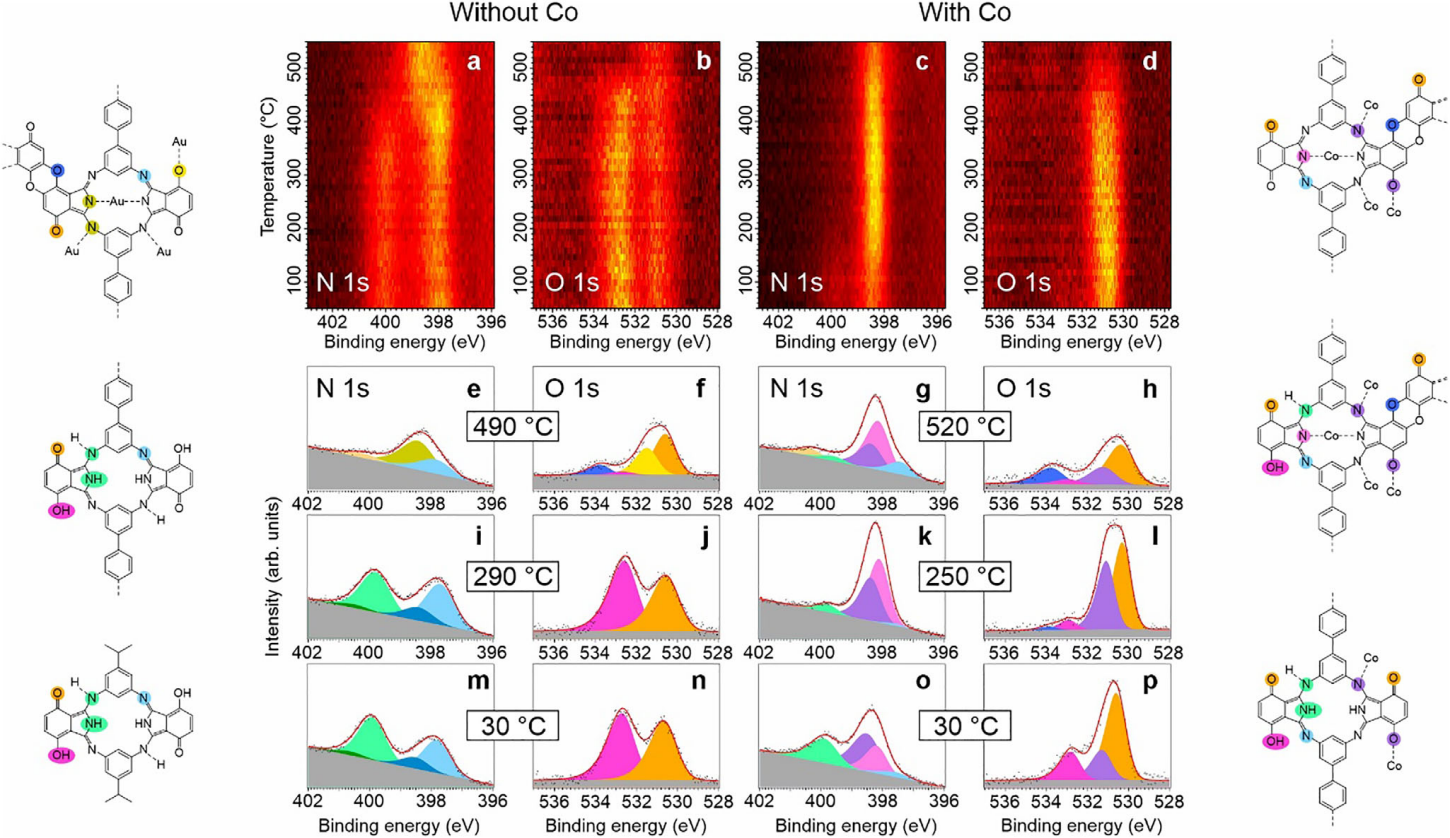
XPS analysis of the system in absence and in presence of co‐deposited cobalt atoms. (a–d) TP‐XPS maps of N 1s and O 1s core levels acquired during the annealing of **1** from RT to 550°C on Au(111) in absence (a and b) and in presence (c and d) of Co (deposited on the surface held at RT after annealing molecule **1** to 290°C, that is, beyond the polymerization temperature). (e–p) HR‐XPS spectra of the N 1s and O 1s core levels acquired after annealing the sample to key temperatures (indicated in the panels). Data acquired using synchrotron radiation with photon energy of 650 eV for all TP‐XPS data and HR‐XPS of O 1s core level, while photon energy of 500 eV was used for the HR‐XPS of N 1s core level. Pass energy (PE) of 20 eV and 10 eV were used for the TP‐XPS maps of N 1s and O 1s, respectively, while PE = 10 eV was used for all HR‐XPS data. Molecular schemes reported in the left and right part of the figure display the types of binding motifs discussed in the text. The colored circles that highlight some of the atoms indicate specific chemical environments and are color‐coded to match the fitting components in the HR‐XPS spectra of panels e‐p.

To unveil the effect of cobalt atoms in the metalation and cyclization processes, we prepared a sample by RT deposition of molecule **1**, annealing to 290°C, and subsequent deposition of Co on the sample held at RT. For this type of sample, we then acquired TP‐XPS maps (Figure 4c,d) and HR‐XPS spectra at selected temperatures. N 1s and O 1s HR‐XPS spectra of the as‐prepared sample (Figure 4o,p) reveal marked differences as compared to the sample in absence of cobalt. The aminic component of the N 1s signal (399.9 eV) is now relatively less intense with respect to the sample without cobalt, indicating a partial metalation of the molecular cores with Co atoms. The iminic component at 397.6 eV is also strongly suppressed and two new components arise at 398.2 and 398.5 eV, which we assign to Co‐bound nitrogen atoms in the center or periphery of the macrocycle, respectively. The O 1s signal shows a concomitant reduction of the ─OH component, presumably due to an enhanced dehydrogenation of the hydroxyl groups operated by adjacent cobalt atoms. We also observe a new component at 531.3 eV that is assigned to the interaction of the dehydrogenated O atoms with cobalt, in agreement with the STM/nc‐AFM observations described above. It is worth noting that the coordination environment of Co atoms might depend on the experimental conditions: while STM/nc‐AFM measurements performed at low temperature (∼4 K) predominantly detect O···Co···O coordination motifs, XPS measurements carried out at room temperature suggest that both oxygen and nitrogen atoms are affected by the presence of cobalt atoms. Annealing this sample to 250°C does not significantly alter the picture, apart from an increased Co‐metalation at the macrocycle centers (Figure 4k) and at the C═O sites, but a new component is observed for the O 1s core level at 533.9 eV (Figure 4l), assigned to the C–O–C bridges. Such component becomes more intense after annealing the sample to 520°C. At this temperature we observe an overall signal reduction for both O 1s and N 1s that may suggest molecular decomposition and desorption. A new N 1s component at 400.36 eV is also visible, which we again assigned to lateral fusion between molecular fragments. The catalytic role of co‐deposited cobalt atoms is underscored by the enhanced dehydrogenation of hydroxyl groups and the observation of the C–O–C fingerprint at lower temperature (∼ 470°C) with respect to the case without cobalt (as evidenced by the comparison of the two TP‐XPS maps in Figures 4b,d). This dual reactivity underscores the tunability of carbaporphyrinoid polymers at interfaces, where the balance between covalent and coordination chemistry provides versatile pathways to engineer multifunctional low‐dimensional materials.

## 3 Conclusion

In conclusion, we have demonstrated a versatile on‐surface strategy to engineer oxygen‐mediated connectivity in carbaporphyrinoid polymers with atomic precision. Starting from hydroxyl‐functionalized dicarbahemiporphyrazine precursors on Au(111), controlled thermal activation induces a surface‐assisted [3+3] cycloaromatization, yielding one‐dimensional polymers that retain reactive hydroxyl termini. From this well‐defined intermediate, two distinct and selectively addressable reaction pathways emerge. Thermal annealing promotes intermolecular dehydrogenative C–O–C coupling between adjacent polymer chains, leading to the formation of dibenzo‐p‐dioxane bridges that covalently interconnect carbaporphyrinoid macrocycles. Alternatively, cobalt deposition redirects the system toward metal–organic architectures, where two‐fold O···Co···O coordination motifs coexist with Co‐metalated macrocyclic cores, giving rise to extended supramolecular networks.

High‐resolution STM and nc‐AFM provide direct real‐space visualization of both covalent O‐heterocyclic linkages and coordination‐driven assemblies, while DFT calculations elucidate the underlying reaction mechanisms and energetic landscapes governing these transformations. Complementary XPS measurements confirm the distinct chemical fingerprints associated with hydroxyl dehydrogenation, O‐heterocycle formation, Au self‐metalation, and cobalt‐assisted coordination, establishing a coherent picture of the thermally and chemically driven surface reactivity. Overall, this work highlights hydroxyl groups as powerful multifunctional handles for directing on‐surface chemistry, providing a basis for extending such reactivity to other molecular scaffolds.

## Author Contributions

**Alberto Martínez‐bajo**: investigation. **Maxence Urbani**: investigation. **Ana Barragán**: investigation. **Óscar Jover**: investigation, formal analysis. **Elena Pérez‐Elvira**: investigation. **Ilaria Tomei**: investigation. **Claudio Goletti**: investigation. **Alejandro Jiménez‐Martín**: investigation. **Aurelio Gallardo**: investigation. **Koen Lauwaet**: investigation. **David Écija**: writing – review, investigation, resources, funding acquisition, and editing. **Diego Soler‐Polo**: investigation, formal analysis, writing – review and editing, writing – original draft, software. **Marco Di Giovannantonio**: writing – original draft, writing – review and editing, formal analysis, supervision, investigation, methodology, funding acquisition. **Tomás Torres**: conceptualization, writing – review and editing, supervision, resources, funding acquisition. **José I. Urgel**: investigation, funding acquisition, formal analysis, supervision, resources, project administration, writing – review and editing, writing – original draft, conceptualization.

## Conflicts of Interest

The authors declare no conflict of interest.

## Supporting information




**Supporting File**: anie73926‐sup‐0001‐SuppMat.docx.

## Data Availability

The data that support the findings of this study are available from the corresponding author upon reasonable request.
