# Supplementary material for: Tunable Oxygen Connectivity in Carbaporphyrinoid Polymers on Metal Surfaces
Source: Angew Chem Int Ed Engl. 2026 Aug 5;65(40):e9635327. doi: 10.1002/anie.9635327 (PMC13618297; doi:10.1002/anie.9635327)
Supplement: Supplementary file 1 [file ANIE-65-e9635327-s001.docx]

**Supporting Information**

**Tunable Oxygen Connectivity in Carbaporphyrinoid Polymers on Metal Surfaces**

Alberto Martínez-Bajo,^[a]^ Maxence Urbani,^[a]^ Ana Barragán,^[a]^ Óscar Jover,^[b]^ Elena Pérez-Elvira,^[a]^ Ilaria Tomei,^[b,c]^ Claudio Goletti,^[c]^ Alejandro Jiménez-Martín,^[a]^ Aurelio Gallardo,^[a]^ Koen Lauwaet,^[a]^ David Écija,^[a,d]^ Diego Soler-Polo,*^,[a]^ Marco Di Giovannantonio,*^, [b]^ Tomás Torres*^,[a,e]^ and José I. Urgel*^,[a,d]^

[a]	Dr. Alberto Martínez-Bajo, Dr. Maxence Urbani, Dr. Ana Barragán, Elena Pérez-Elvira, Dr. Alejandro Jiménez Martín, Dr. Aurelio Gallardo, Dr. Koen Lauwaet, Prof. David Écija, Prof. Tomás Torres and Dr. José I. Urgel
IMDEA Nanoscience, C/ Faraday 9, Campus de Cantoblanco, 28049 Madrid, Spain
E-mail: diego.soler@imdea.org, jose-ignacio.urgel@imdea.org

[b]	Dr. Óscar Jover, Ilaria Tomei and Dr. Marco Di Giovannantonio
CNR – Istituto di Struttura della Materia (CNR-ISM), 00133 Roma, Italy

E-mail: marco.digiovannantonio@cnr.it

[c]	Ilaria Tomei and Prof. Claudio Goletti

Dipartimento di Fisica, University of Rome “Tor Vergata”, 00133 Roma, Italy

[d]	Prof. David Écija and Dr. José I. Urgel

Unidad de Nanomateriales avanzados, Imdea Nanoscience, Unidad asociada al CSIC por el ICMM, 28049 Madrid, Spain

[e]	Prof. Tomás Torres

Departamento de Química Orgánica and Institute for Advanced Research in Chemistry (IAdChem), Universidad Autónoma de Madrid, 28049 Madrid, Spain

E-mail: [tomas.torres@uam.es](mailto:tomas.torres@uam.es)

**Table of Contents**

1. **Experimental and computational methods**
2. **Synthesis and characterization of dicarbahemiporphyrazine 1**
3. **On-surface synthesis**
4. **References**

**1. Experimental and computational methods**

*1.1 Experimental details*

The experiments were conducted at IMDEA Nanoscience (Madrid, Spain) in a custom-designed ultra-high vacuum system with a base pressure below 2.0 × 10^−10^ mbar, which hosts a commercial low-temperature microscope with STM/AFM capabilities from Scienta Omicron. The Au(111) substrate was prepared by sputtering Ar^+^ (E = 1.5 keV) followed by annealing at 740 K for 10 minutes. The molecular precursor **1** was deposited on the clean Au(111) surface using thermal evaporation (Kentax TCE-BSC. T_sublimation_ = 240 ºC) with a typical deposition rate of 0.5 Å/min at room temperature, controlled by a quartz microbalance (LewVac). After deposition of the precursor, the sample was annealed at 280 °C for 10 minutes to promote the [3+3] cycloaromatization reaction, yielding the one-dimensional hydroxyl terminated polymers, **2**. The one-dimensional polymers, **2**, can be evolved into two different metalorganic frameworks, **3** and **4**, with two different treatments. Performing an annealing at 315 ºC to a sample containing **2** yields the two-dimensional dioxin polymers with Au metalation within the dcHp cavities, **3**. On the other hand, cobalt deposition, with the use of a homemade e-beam evaporator, over a sample containing **2** held at 130 ºC to promote the molecular diffusion along the substrate, yields a metalorganic framework containing Co metalation between the hydroxyl groups of different monomers of the 1D chains and within the dcHp cavity, **4**.

All temperatures reported for the STM/AFM experiments were measured using a pyrometer (CellaTemp PA 28 AF 10/D from Keller). These measurements correspond to a sample treatment in which the temperature was increased from room temperature (RT) to the target value, held constant for 10 minutes, and then cooled back to RT. In contrast, the temperatures reported for the HR-XPS measurements correspond to a flash-annealing procedure, in which the sample temperature was ramped at a constant heating rate of 4 °C/min up to the target temperature and then immediately cooled to RT. This difference in thermal treatment accounts for the apparent discrepancies between the reported temperatures, with the STM/AFM values appearing lower than those used for XPS. STM images were acquired in either constant-current or constant height mode with tungsten tips that were electrochemically etched, functionalized with CO, at a sample temperature of 4.3 K (LakeShore). High-resolution STM images were acquired by recording the current channel and nc-AFM images using CO-functionalized tips to scan the polymers in constant-height mode. Non-contact AFM measurements were performed with a tungsten tip attached to a Qplus tuning fork sensor (Scienta Omicron). The tip was a posteriori functionalized by a controlled adsorption of a single CO molecule at the tip apex from a previously CO-dosed surface. The functionalized tip enables the imaging of the intramolecular structure of organic molecules. The sensor was driven at its resonance frequency (~26 kHz for Qplus) with a constant amplitude of ~80 pm. The shift in the resonance frequency of the sensor (with the attached CO-functionalized tip) was recorded in a constant-height mode (Scienta Omicron Matrix electronics and MFLI by Zurich Instruments for Scienta Omicron). d*I*/d*V* signals were obtained using the lock-in technique with a Stanford Research Systems SR830. The figure captions indicate the open feedback settings and subsequent tip distance (Z_offset_) above the STM setpoint for each image. The scanning parameters used for each figure are described in the figure captions. The STM images and STS curves were analyzed using Wavemetrics Igor Pro.

*1.2 XPS results*

High-resolution X-ray photoelectron spectroscopy (HR-XPS) and temperature-programmed XPS (TP-XPS) measurements were performed in situ at the SuperESCA beamline of the Elettra synchrotron radiation facility in Trieste, Italy.^[1]^ The experimental chamber is equipped with a 150 mm Phoibos hemispherical electron energy analyzer (SPECS GmbH), including a home-made large area delay line detector. The spectra were measured in normal emission configuration. HR N 1s and O 1s core levels were recorded with photon energy of 500 eV and 650 eV, respectively. TP-XPS spectra were collected with photon energy of 650 eV in all cases and performed during sample heating from room temperature to 550 °C with a 4 °C/min heating rate. All spectra were energy-aligned with respect to the Fermi level of the gold substrate and normalized to the photon flux. Spectral deconvolution of the HR curves was performed according to a least-squares minimization. Parameters and details are reported in Table S1.

*1.3 Computational details*

To study the reaction that leads from I1 to F3 in Fig. 2, we have employed Fireball/AMBER,^[2]^ a QM/MM method that combines the molecular dynamics code AMBER^[3]^ with Fireball,^[4]^ a pseudo-atomic local orbital-based DFT code that offers an excellent balance between computational performance and accuracy. The relative energies of the different meta-stable states have been further corroborated by means of higher-level DFT calculations with FHI-AIMS.^[5]^

We explore the formation of an O-Heterocylic coupling on one side of the molecule by means of Steered Molecular Dynamics (SMD)^[6]^ and Jarzynski's equation.^[7]^ We divide such process in the three stages described in the main text: 1) Dehydrogenation of O atom (see Fig 2, structures I1 and F1 in Fig 2)) assisted by an adatom, 2) Formation of the C1-O-C2 bridge (see structures I2 and F2) and 3 in Fig 2)) Final dehydrogenation of the C2 carbon assisted by an adatom (see structures I3 and F3 in Fig 2).

We use, respectively, reactions coordinates R1 = dist(Au1,H1) – dist(C1,H1), R2 = dist(C1,O) + dist(O,C2) and R3 = dist(Au2,H2) – dist(C2,H2). For each of these reactions, we thermalize the initial states, generate five independent structures at equilibrium using an Umbrella potential and compute a SMD for each of them. For stages 1 and 3 (dehydrogenation), the reaction coordinate is dragged in the SMDs from 1.50 to -0.75 A. For stage 2, the reaction coordinate R2 is dragged in the SMDs from 5.2 to 2.5 A. In all cases we employ 10000 steps of a time step of 0.5 fs for each SMD, We have included RC1, RC2 and RC3 in a single global „reaction progrees“ (RP) parameter in which we normalize the RCs to have the same length and to be ordered in a single axis. The SMD are integrated by means of Jarzynski's equation, a refinement of Thermodynamic Integration that computes Free Energy profiles from work averages of possibly out-of-equilibrium simulations. The resulting overall profile, shown in Fig. 2 shows three local minima (F1,F2 and F3) and three barriers for each for the three stages.

The SMD simulations are carried out for the molecular system sitting on one layer of a 14x8 slab of Au(111).

*1.4 Synthetic procedures and materials*

Synthetic procedures were carried out under an inert argon atmosphere and in dry solvents. All reagents and solvents were used as received. 5- isopropylbenzene-1,3-diamine was previously described and characterised by us in the literature,^[8]^ which was synthetized according to methods reported by Yu *et al*.^[9]^ 2,3-dicyanobenzoquinone was purchased at TCI and 1,2-ethanediol at sigma-Aldrich. All solvents used for purifications (ISO reagent grade), UV-Vis experiments (spectroscopic grade) and deuterated solvents for NMR experiments were purchased at *Sharlab*.

*1.5 Instruments*

- **NMR.** ^1^H (400 MHz) and ^1^H-decoupled ^13^C (100 MHz) NMR spectra were recorded on a Bruker TopSpin AV-400 spectrometer at 298 K. Chemical shifts are reported in parts per million (ppm) relative signal(s) of the partially deuterated solvents as internal standards. Multiplicities are denoted as follows: s = singlet, d = doublet, t = triplet, sept = septet, m = multiplet, and the coupling constants (*J*_H_-*J*_H_) are given in Hz.
- **UV/Vis** absorption spectrum was recorded on an *Agilent* Cary 60 UV-Vis spectrophotometer at room temperature, using a standard 10 mm UV cuvette (QS).
- **MS** experiments were performed by the *Servicio Interdepartamental de Investigacion (SIdI)* at the *Autonoma* University of Madrid. Atmospheric-pressure chemical ionization (APCI) and electrospray ionization (ESI) coupled to a time of flight analyser (QTOF) mass experiments were recorded on a Bruker Maxis II.

**2. Synthesis and characterization of dicarbahemiporphyrazine 1.**

**Scheme S1**. Synthesis of dcHp **1**; reagents and conditions: i) ethane-1,2-diol, reflux (200‒210ºC), 22h (**1**: quantitative).

**Dicarbahemiporphyrazine 1**. A solution of 5-isopropylbenzene-1,3-diamine^[8,9]^ (150.5 mg, 1.00 mmol) and 2,3-dicyanobenzoquinone (160.4 mg, 1.00 mmol) in ethane-1,2-diol (2.5 mL) was degassed by bubbling argon for 15 min. The solution was then let under argon and heated at reflux (200‒210ºC) for 24 h. After cooling to rt, the mixture was diluted in hexanes (*ca.* 75 mL) and the resulting precipitate sonicated vigorously for 5 min. The solid was filtrated off under vacuum over a conic funnel with a paper filter, then triturated and washed several times with EtOH (total of *ca.* 50 mL) till colourless filtrates and air-dried. Finally, the solid was successively triturated and washed several times with MeOH (*ca.* 200 mL), CH_2_Cl_2_ (*ca.* 200 mL), *n*-pentane (*ca.* 200 mL) and air-dried. The sample was then collected in a vial, finely grounded and dried at 70°C under vacuum (*ca.* 1 mbar) for a couple of hours to afford **1** as a dark-black powder (293 mg, 0.50 mmol; Yield: quantitative). dcHp **1** is almost insoluble in all common solvents. However, the tetra sodium salt of dcHp**-1** (**1**^4-^, 4Na^+^) presents a good solubility in MeOH or DMSO solutions (deeply blue-coloured), and therefore NMR and UV-visible spectra have been recorded for the *in situ* formed tetra sodium salt upon addition of NaOD (40% wt. in D_2_O) or NaOH (pellets), respectively. ^1^H NMR (400 MHz, MeOD-d4+one drop of NaOD 40 wt. % in D_2_O, 25°C): δ_H_ = 10.11 (t, ^4^*J*(D,C) = 2Hz, 2H; H-*D*), 6.87 (d, ^4^*J*(C,D) = 2Hz, 4H; H-*C*), 6.67 (s, 4H; H-*E*), 2.93 (sept, ^3^*J*(a,b) = 6.9 Hz, 2H; H-b), 1.32 ppm (d, ^3^*J*(a,b) = 6.9 Hz, 12H; H-a). ^13^C NMR (100 MHz, MeOD-d4+one drop of NaOD 40 wt. % in D_2_O, 25°C) δ_C_= 165.3, 165.1, 150.6, 140.0, 134.5, 113.1, 109.9, 108.4, 33.8 (*C*H), 22.8 ppm (*C*H_3_). UV/Vis (MeOH+NaOH; 25°C): λ_max_ (ε) = 614 (76 800), 570 (35 040), 389 (br. 17 700), 371 (18 800), 322 (13 500), 266 nm (33 400 dm^3^ mol^‒1^ cm^–1^). APCI-HRMS of neutral form (**1**) (neg. mode; matrix: CH_2_Cl_2_) *m/z* (%) found: 566.1001 (29) [M-OH-3H]^1‒^, 585.2238 (100) [M-H]^1‒^; *m/z* calcd for C_34_H_29_N_6_O_4_: 585.2256; found 585.2238 [M-H]^‒^. ESI-HRMS of the tetra sodium salt [**1**^4-^, 4Na^+^] (negative mode; matrix: MeOH) *m/z* (%) found: 292.1097 (54.7) [M-4Na+2H]^2‒^, 585.2266 (100) [M-4Na+3H]^1‒^, 607.2089 (12) [M-3Na+2H]^1‒^; *m/z* calcd for for C_34_H_29_N_6_O_4_: 585.2242; found: 585.2266 [M-4Na+3H]^1‒^ and for C_34_H_28_N_6_NaO_4_: 607.2075; found 607.2089 [M-3Na+2H]^1‒^.

**Figure S1**. Pictures of dcHp-**1**; from left to right: (A) dry solid, (B) in MeOH (insoluble), (C) in MeOH + 1 drop of NaOH 0.1 M in H_2_O (*in situ* formed [(dcHp-**1**^4-^), 4 Na^+^]).

**3. NMR, MS, and UV-vis spectra of the tetra sodium salt of dcHp-1 (1^4-^, 4Na^+^).**

**Figure S2.** **^1^**H NMR (MeOD-d4 + 1 drop of NaOD 40% wt. in D_2_O, 400 MHz) of the tetra sodium salt of dcHp**-1** (**1**^4-^, 4Na^+^) (* denote the residual methanol and water peaks from the MeOD-d4 and D_2_O solvents; ** denotes a signal from residual ethane-1,2-diol, the solvent of reaction).

**Figure S3.** 2D-COSY 45 ^1^H/^1^H NMR partial spectrum (MeOD-d4 + 1 drop of NaOD 40%wt. in D_2_O, 400 MHz) of the tetra sodium salt of dcHp**-1** (**1**^4-^, 4Na^+^).

**Figure S4.** ^13^C NMR (MeOD-d4 + 1 drop of NaOD 40% wt. in D_2_O, 100 MHz) of the tetra sodium salt of dcHp**-1** (**1**^4-^, 4Na^+^) (* denote the residual methanol signal from the MeOD-d4 solvent; ** denotes a signal from residual ethane-1,2-diol, the solvent of reaction).

*Fragmentation

[M-(OH)±nH]^1‒^

[M-H] ^1‒^

**Figure S5.** APCI-HRMS (negative mode; matrix: CH_2_Cl_2_) spectrum of dcHp**-1** (* denotes a fragmentation peak, see MS/MS experiment on next figure).

[M-(OH)-2H]^1‒^

[M-(OH)+H]^1‒^

[M-(OH)]^1‒^

MS/MS at *m/z* =585.21 Da

**Figure S6.** APCI-MS/MS experiment at *m/z* = 585.21 (negative mode; matrix: CH_2_Cl_2_) of dcHp**-1.**

**Simulated**

Isotopic distribution simulated

for C_34_H_29_N_6_O_4_ ([M-H]^1−^)

**Experimental**

[M-H] ^1‒^

**Figure S7.** Experimental (top inset) APCI-HRMS spectrum (negative mode; matrix: CH_2_Cl_2_) of dcHp**-1**

and simulated isotopic distribution profile for [M-H]^1-^ (bottom inset).

**Figure S8.** UV/Visible absorption spectrum of the tetra sodium salt of dcHp**-1** (**1**^4-^, 4Na^+^) in MeOH solution.

**3. On-surface synthesis**

**Figure S9.** Polymer **2** and its stabilization by O···H interactions. a) Constant-height STM image of a segment of **2** acquired with a CO-functionalized tip. V_b_ = 5 mV, I_t_ = 50 pA, scale bar: 1 nm. b) Same STM image as the one depicted in (a) with the structural model superimposed. c) Augmented structural model highlighting in red the formation of O···H bonds between adjacent polymers.

**Figure S10.** Statistical analysis of the chain-length distributions for all the polymeric species (**2**, **3**, **3′** and **4**). Polymers **2** and **3** exhibit comparable length distributions within experimental uncertainty (orange bar), indicating that the gold self-metalation does not significantly alter the average chain length, whereas differences emerge upon O-heterocyclic coupling and cobalt metalation and coordination. Statistics out of 130 molecular units.

**Figure S11.** Polymer **2** (before) and polymer **4** (after) cobalt metalation and coordination. a) Constant-current STM and zoomed-in nc-afm images of a segment of **2** acquired with a CO-functionalized tip, together with the corresponding atomistic model. Constant-current STM image: V_b_ = 100 mV, I_t_ = 120 pA, scale bar: 2 nm; nc-afm image: Z-offset 130 pm below STM set point: 5 mV, 50 pA. b) Same as in (a) for a segment of 4 after Co deposition. V_b_ = 100 mV, I_t_ = 50 pA, scale bar: 1 nm; nc-afm image: Z-offset 120 pm below STM set point: 5 mV, 50 pA. Apart from the clear differences between **2** and **4** observed at the dcHp macrocycles, nc-afm images show for **2** relatively localized contrast at the hydroxyl functionalities appearing as distinct protrusions at the periphery, consistent with non-covalent interchain interactions and the absence of strong lateral linking motifs. In contrast, **4** exhibits a more continuous and periodically modulated contrast along the chain, which is attributed to the presence of Co-mediated O···Co···O coordination bridges.

**Figure S12.** Zero-bias feature assigned to a Kondo resonance state in **4**. a) Constant-height STM image of a segment of **4** acquired with a CO-functionalized tip. V_b_ = 5 mV, I_t_ = 50 pA, scale bar: 1 nm. b) d*I*/d*V* spectra acquired on the Au(111) clean substrate (orange line) and at the center of a dcHp unit shown in panel (a): Co-metalated center (blue) and metal-free center (red). Open feedback parameters for d*I*/d*V* spectra: V_b_ = 50 mV, I_t_ = 250 pA, V_rms_ = 3 mV.

*Bond vibration in the O-N-H hydrogen bond*

The H atoms in the O are close to the N in the outer ring of the hemiporphyrazine, and so they oscillate along the resulting hydrogen bond. Long ab initio free molecular dynamics (performed with the software Fireball) of 30 ps shows that the H-O covalent bond yields a minimum energy (left extreme in Fig. S11), but the opposite configuration, when the H is covalently bounded to the N, is metastable and the energy difference is extremely small. The free energy profile for this hydrogen bond deviates from the simple double-well potential, presenting quasi-metastable states in intermediate positions for the H, see Fig. S11 below.

**Figure S13** Free energy profile for a hydrogen bond. Configurations with a covalent O-H are the most stable, but the complementary covalent N-H also is significantly populated.

The given barriers of around 110 meV (from left to right in Fig. S11) or 90 meV (from right to left in Fig. S11) yield half-life times of between 1 to 7 ps at RT. Whereas this timescale is still small for the QM/MM simulations, it makes clear that in experimental conditions the hydrogen will appear delocalized between the N and H atoms. This rationalizes the observed features in the XPS data shown in Figure 4.

|  |  | Binding energy  (eV) | FWHM  (eV) | Relative amount  (%) |
| --- | --- | --- | --- | --- |
| Without Co | N 1s (30 °C) | 397.82 | 1.02 | 36 |
|  |  | 398.52 | 1.02 | 13 |
|  |  | 399.93 | 1.02 | 46 |
|  |  | 400.63 | 1.02 | 5 |
|  | N 1s (290 °C) | 397.71 | 1.10 | 41 |
|  |  | 398.41 | 1.10 | 14 |
|  |  | 399.81 | 1.10 | 41 |
|  |  | 400.51 | 1.10 | 4 |
|  | N 1s (490 °C) | 397.87 | 1.18 | 30 |
|  |  | 398.44 | 1.18 | 60 |
|  |  | 400.20 | 1.18 | 10 |
|  | O 1s (30 °C) | 530.83 | 1.51 | 45 |
|  |  | 532.94 | 1.51 | 55 |
|  | O 1s (290 °C) | 530.58 | 1.50 | 44 |
|  |  | 532.59 | 1.50 | 56 |
|  | O 1s (490 °C) | 530.60 | 1.21 | 50 |
|  |  | 531.50 | 1.21 | 33 |
|  |  | 532.70 | 1.21 | 5 |
|  |  | 533.80 | 1.21 | 12 |
| With  Co | N 1s (30 °C) | 397.60 | 1.02 | 6 |
|  |  | 398.20 | 1.02 | 24 |
|  |  | 398.53 | 1.02 | 43 |
|  |  | 399.92 | 1.02 | 27 |
|  | N 1s (250 °C) | 397.60 | 0.76 | 2 |
|  |  | 398.12 | 0.76 | 54 |
|  |  | 398.39 | 0.76 | 37 |
|  |  | 399.80 | 0.76 | 7 |
|  | N 1s (520 °C) | 397.42 | 0.84 | 13 |
|  |  | 398.16 | 0.84 | 49 |
|  |  | 398.39 | 0.84 | 25 |
|  |  | 399.62 | 0.84 | 5 |
|  |  | 400.36 | 0.84 | 8 |
|  | O 1s (30 °C) | 530.60 | 1.10 | 60 |
|  |  | 531.30 | 1.10 | 21 |
|  |  | 532.82 | 1.10 | 19 |
|  | O 1s (250 °C) | 530.32 | 0.98 | 51 |
|  |  | 531.10 | 0.98 | 40 |
|  |  | 532.95 | 0.98 | 6 |
|  |  | 533.89 | 0.98 | 3 |
|  | O 1s (520 °C) | 530.38 | 1.47 | 50 |
|  |  | 531.24 | 1.47 | 22 |
|  |  | 532.96 | 1.47 | 7 |
|  |  | 533.81 | 1.47 | 21 |

**Table S1.** Fitting parameters used for the deconvolution of the HR-XPS curves in Figure 4 of the main text. For each fitting component, the center position, full width at half-maximum (FWHM) and relative amount are provided. A linear background and Voigt functions (Gaussian:Lorentzian ratio of 90:10) have been used in all cases. The FWHM values have been constrained to be the same within the fit of a single curve.

**4. References**

[1]	A. Abrami, M. Barnaba, L. Battistello, A. Bianco, B. Brena, G. Cautero, Q. H. Chen, D. Cocco, G. Comelli, S. Contrino, F. DeBona, S. Di Fonzo, C. Fava, P. Finetti, P. Furlan, A. Galimberti, A. Gambitta, D. Giuressi, R. Godnig, W. Jark, S. Lizzit, F. Mazzolini, P. Melpignano, L. Olivi, G. Paolucci, R. Pugliese, S. N. Qian, R. Rosei, G. Sandrin, A. Savoia, R. Sergo, G. Sostero, R. Tommasini, M. Tudor, D. Vivoda, F.-Q. Wei, F. Zanini, “Super ESCA: First beamline operating at ELETTRA” *Rev. Sci. Instrum.* **1995**, *66*, 1618–1620.

[2]	J. I. Mendieta-Moreno, R. C. Walker, J. P. Lewis, P. Gómez-Puertas, J. Mendieta, J. Ortega, “fireball/amber: An Efficient Local-Orbital DFT QM/MM Method for Biomolecular Systems” *J. Chem. Theory Comput.* **2014**, *10*, 2185–2193.

[3]	D. A. Case, T. E. Cheatham III, T. Darden, H. Gohlke, R. Luo, K. M. Merz Jr., A. Onufriev, C. Simmerling, B. Wang, R. J. Woods, “The Amber biomolecular simulation programs” *J. Comput. Chem.* **2005**, *26*, 1668–1688.

[4]	J. P. Lewis, P. Jelínek, J. Ortega, A. A. Demkov, D. G. Trabada, B. Haycock, H. Wang, G. Adams, J. K. Tomfohr, E. Abad, H. Wang, D. A. Drabold, “Advances and applications in the FIREBALL ab initio tight-binding molecular-dynamics formalism” *Phys. Status Solidi B* **2011**, *248*, 1989–2007.

[5]	V. Blum, R. Gehrke, F. Hanke, P. Havu, V. Havu, X. Ren, K. Reuter, M. Scheffler, “Ab initio molecular simulations with numeric atom-centered orbitals” *Comput. Phys. Commun.* **2009**, *180*, 2175–2196.

[6]	S. Izrailev, S. Stepaniants, B. Isralewitz, D. Kosztin, H. Lu, F. Molnar, W. Wriggers, K. Schulten in *Comput. Mol. Dyn. Chall. Methods Ideas* (Eds.: P. Deuflhard, J. Hermans, B. Leimkuhler, A.E. Mark, S. Reich, R.D. Skeel), Springer, Berlin, Heidelberg, **1999**, pp. 39–65.

[7]	C. Jarzynski, “Nonequilibrium Equality for Free Energy Differences” *Phys. Rev. Lett.* **1997**, *78*, 2690–2693.

[8]	A. Barragán, M. Urbani, A. Gallardo, E. Pérez-Elvira, Ó. Jover, K. Lauwaet, J. M. Gallego, R. Miranda, M. D. Giovannantonio, D. Écija, T. Torres, J. I. Urgel, “On-Surface Synthesis of Covalently-Linked Carbaporphyrinoid-Based Low-Dimensional Polymers” *Small* **2025**, *21*, 2408085.

[9]	L. Yu, M. Huang, T. Xu, L. Tong, X. Yan, Z. Zhang, Y. Xu, C. Yun, H. Xie, K. Ding, X. Lu, “A structure-guided optimization of pyrido[2,3-*d*]pyrimidin-7-ones as selective inhibitors of EGFRL858R/T790M mutant with improved pharmacokinetic properties” *Eur. J. Med. Chem.* **2017**, *126*, 1107–1117.
